# Symptom Burden, Depressive Symptoms, Resilient Coping, and Pretreatment Health-Related Quality of Life with Breast, Gynecological and Genitourinary Cancer

**DOI:** 10.3390/curroncol33080482

**Published:** 2026-08-15

**Authors:** Caterina Calderon, Patricia Cruz-Castellanos, Asia Ferrandez-Arias, Lucía Roncero-Sánchez, Valle Rodríguez-Morón, Rocío Galán-Moral, David Gómez-Sánchez, María Nevado-Rodríguez, Marina Gustems, Paula Jiménez-Fonseca

**Affiliations:** 1 Department of Clinical Psychology and Psychobiology, University of Barcelona, Passeig de la Vall d’Hebron 171, 08035 Barcelona, Barcelona, Spain; 2 Department of Medical Oncology, Hospital General Universitario de Ciudad Real, 13005 Ciudad Real, Ciudad Real, Spain; 3 Department of Medical Oncology, Hospital General Universitario de Elche, 03203 Elche, Alicante, Spain; 4 Department of Medical Oncology, Hospital Universitario Central de Asturias, ISPA, 33011 Oviedo, Asturias, Spain; 5 Department of Medical Oncology, Hospital Universitario de Canarias, 38320 San Cristóbal de La Laguna, Santa Cruz de Tenerife, Spain; 6 Department of Medical Oncology, Hospital Universitario de Navarra, 31008 Pamplona, Navarra, Spain; 7 Department of Medical Oncology, Hospital Universitario Morales Meseguer, 30008 Murcia, Murcia, Spain

**Keywords:** patient-reported outcomes, health-related quality of life, symptom burden, depressive symptoms, resilient coping, supportive oncology, breast cancer, gynecological cancer, genitourinary cancer

## Abstract

Pretreatment health-related quality of life (HRQoL) may identify supportive care needs, but the joint contribution of symptom burden, depressive symptoms, and resilient coping remains insufficiently characterized. This multicenter baseline analysis included 194 adults with breast (*n* = 86), gynecological (*n* = 77), or genitourinary (*n* = 31) cancer from six Spanish hospitals before systemic therapy. Global HRQoL was assessed with the EuroQol visual analogue scale EQ-VAS; symptom burden with EORTC QLQ-C30 symptom scales/items; depressive symptoms with the Brief Symptom Inventory-18 depression subscale; and resilient coping with the Brief Resilient Coping Scale. EQ-VAS scores did not differ by tumor site (*p* = 0.369). Symptom burden was strongly inversely associated with HRQoL (r = −0.827, *p* < 0.001). In the moderated mediation model, higher symptom burden was associated with higher depressive symptoms (B = 0.275, 95% CI 0.118 to 0.431); symptom burden (B = −0.642, 95% CI −0.715 to −0.569) and depressive symptoms (B = −0.529, 95% CI −0.743 to −0.316) were associated with lower HRQoL scores. Indirect effects through depressive symptoms were statistically different from zero, but resilient coping did not significantly moderate this indirect association. These cross-sectional findings support pretreatment screening for symptoms and depressive symptoms to guide early supportive oncology care.

## Simple Summary

Patients starting systemic cancer treatment often report symptoms and emotional distress that may affect perceived health before therapy begins. In this multicenter Spanish study, we assessed 194 patients with breast, gynecological, or genitourinary cancer before systemic treatment. Patient-reported symptom burden and depressive symptoms were more strongly associated with global health-related quality of life than tumor site, stage, treatment type, or performance status. Depressive symptoms statistically accounted for part of the association between symptom burden and quality of life, whereas resilient coping did not significantly reduce this pathway. These findings support early pretreatment screening of symptoms and emotional distress and reinforce the need to integrate symptom control and psycho-oncology within supportive care pathways from the start of systemic therapy.

## 1. Introduction

Health-related quality of life (HRQoL) and patient-reported outcomes (PROs) have moved from optional ancillary endpoints to integral components of cancer care. They capture how disease and treatment affect symptoms, functioning, and perceived health, complementing clinician-assessed toxicity, imaging response, and survival endpoints [1]. In breast, gynecological, and genitourinary cancers, HRQoL assessment is particularly relevant because disease and treatment may affect fatigue, pain, urinary and bowel function, sexual functioning, body image, fertility concerns, and social participation [2,3]. Although these tumor groups differ in sex distribution and symptom profiles, they share important supportive care needs before systemic therapy. Because these are not fully captured by conventional clinical indicators, systematic pretreatment assessment may help identify needs and enable supportive interventions before treatment-related toxicities further compromise quality of life.

Symptom burden is one of the most consistent correlates of HRQoL in oncology. Patients with breast, gynecological, and genitourinary cancers frequently experience multiple concurrent symptoms, including fatigue, pain, sleep disturbance, gastrointestinal symptoms, urinary problems, appetite loss, sexual dysfunction, and functional limitations [2,3]. Because symptoms often co-occur and may interact with daily functioning and emotional well-being, an aggregate patient-reported symptom burden score may provide a clinically interpretable summary of vulnerability before treatment begins.

Depressive symptoms are also central to pretreatment supportive care assessment. Depression and depressive symptomatology are associated with global quality of life, emotional and social functioning, treatment adaptation, toxicity experience, and longitudinal distress trajectories in cancer populations [4,5,6]. From a clinical perspective, depressive symptoms should not be viewed as a psychosocial outcome separate from physical symptoms. Instead, they may represent a statistical pathway linking symptom burden and global HRQoL.

Resilience has been proposed as a protective resource in cancer and is conceptualized in the present study as the tendency to respond adaptively to stress, reflecting an adaptive coping style rather than a broad multidimensional construct. Higher resilience or resilient coping has been associated with better HRQoL and lower psychological distress in breast, gynecological, and genitourinary cancers [7,8,9,10]. However, whether resilient coping buffers the association between symptom burden and depressive symptoms at the start of systemic therapy remains uncertain. However, it remains unclear whether resilient coping attenuates the association between symptom burden and depressive symptoms at the start of systemic therapy. From this perspective, resilient coping was hypothesized to moderate this association. Clarifying this relationship may help identify patients who remain vulnerable despite adaptive coping resources. Together, these factors may interact shape patients’ perceptions of their health before treatment initiation.

Addressing these gaps may improve early identification of patients requiring supportive oncology interventions before treatment begins. First, many studies have evaluated symptoms, depressive symptoms, coping resources, and HRQoL in isolation or within single tumor types. Second, fewer studies have examined an integrated model that includes both a statistical indirect pathway through depressive symptoms and a potential moderating role of resilient coping. Third, evidence is limited at the pretreatment systemic therapy visit, when supportive care needs are clinically actionable and when baseline patient-reported vulnerability may inform monitoring intensity.

The present study extends previous work by integrating symptom burden, depressive symptoms, resilient coping, and global HRQoL within a single pretreatment model in patients with breast, gynecological, and genitourinary cancers. The objectives were to describe global HRQoL according to sociodemographic and clinical characteristics; to examine associations among symptom burden, depressive symptoms, resilient coping, and HRQoL; and to test a first-stage moderated mediation model in which depressive symptoms statistically account for the association between symptom burden and HRQoL, while resilient coping moderates the association between symptom burden and depressive symptoms. We hypothesized that greater symptom burden would be associated with poorer HRQoL directly and indirectly through higher depressive symptoms, and that higher resilient coping would attenuate the association between symptom burden and depressive symptoms. Because this analysis used baseline data only, all pathways were interpreted as cross-sectional statistical associations rather than temporal or causal mechanisms.

## 2. Materials and Methods

### 2.1. Study Design and Setting

This manuscript reports a baseline cross-sectional analysis from a multicenter prospective observational cohort conducted by the NEOteam research group, and the Department of Psychology of the University of Barcelona. Recruitment took place between May 2025 and January 2026 in medical oncology departments from six Spanish hospitals. The present analysis was restricted to pretreatment data collected before initiation of systemic anticancer therapy. Reporting was structured in accordance with the Strengthening the Reporting of Observational Studies in Epidemiology (STROBE) guidance where applicable [11].

### 2.2. Participants and Recruitment

The present study represents a secondary analysis of the baseline assessment from an ongoing multicenter prospective cohort; therefore, all consecutive eligible patients recruited during the study period were included. Eligible participants were adults aged 18 years or older with histologically confirmed breast, gynecological, or genitourinary cancer for whom systemic anticancer therapy was planned. The treating oncologist assessed each patient’s eligibility during routine clinical care. Participants were required to be able to understand the study information, provide written informed consent, and complete the self-administered questionnaires. Exclusion criteria were a diagnosis of another advanced cancer within the previous two years and clinically evident cognitive, communication, or functional limitations considered likely to compromise the validity of self-reported data. No formal cognitive screening instrument was administered. Potentially eligible patients were identified consecutively after the systemic treatment plan had been established and were invited to participate. All participants provided written informed consent before completing the study assessments. Of the 207 patients screened for eligibility, 194 met the inclusion criteria and were included in the present analysis, while 13 were excluded because they met one or more prespecified exclusion criteria.

The study protocol was approved by the Research Ethics Committee of Asturias and by the research ethics committees of participating institutions (protocol code IRB00003099; approval date 12 May 2025). All procedures were conducted in accordance with the Declaration of Helsinki and applicable national regulations.

### 2.3. Sociodemographic and Clinical Variables

Baseline sociodemographic variables included sex (male vs. female), age (<65 vs. ≥65 years), marital status (partnered vs. not partnered), educational level (primary school or lower vs. secondary education or higher), and employment status (employed vs. unemployed/retired). The age cutoff of 65 years was retained as a conventional threshold in oncology and geriatric stratification. Clinical variables were extracted from medical records and included tumor site (breast, gynecological, or genitourinary), histology (adenocarcinoma vs. other), resectability (resectable vs. unresectable), disease stage (locally advanced vs. metastatic), planned systemic treatment (chemotherapy alone, chemotherapy plus immunotherapy, chemotherapy plus targeted therapy, or other regimens), Eastern Cooperative Oncology Group performance status (ECOG-PS; 0–1 vs. ≥2), and oncologist-estimated life expectancy (<18 vs. ≥18 months).

### 2.4. Patient-Reported Measures

Global HRQoL/self-rated health was assessed using the EuroQol visual analogue scale (EQ-VAS), a 0–100 scale on which higher scores indicate better self-rated health. Spanish reference data and validity evidence support the use of the EQ-5D-5L system and EQ-VAS in the Spanish population [12]. The EQ-VAS was used as the HRQoL outcome, whereas symptom burden was derived from the EORTC QLQ-C30 symptom items, thereby minimizing conceptual overlap study variables.

Depressive symptoms were assessed with the depression subscale of the Brief Symptom Inventory-18 (BSI-18). Raw scores were converted to standardized T-scores following the instrument manual [13], with higher scores indicating greater depressive symptom severity. The BSI-18 has been widely used in oncology, and the Spanish version has shown adequate psychometric properties in patients with cancer [13,14]. The internal consistency of the BSI-18 depression subscale in the present sample was satisfactory (Cronbach’s α = 0.80).

Patient-reported symptom burden was derived from the European Organization for Research and Treatment of Cancer Quality of Life Questionnaire Core 30 (EORTC QLQ-C30) symptom scales and single items, following standard EORTC scoring procedures. Scores were transformed to a 0–100 metric, with higher values indicating greater symptom burden [15,16]. The internal consistency of the symptom burden score was good in the present sample (Cronbach’s α = 0.83).

Resilient coping was assessed with the Brief Resilient Coping Scale (BRCS), a four-item instrument that assess the tendency to cope with stress adaptively. Higher scores indicating greater resilient coping. The Spanish version has demonstrated adequate measurement properties in cancer populations [17,18]. The BRCS showed good internal consistency in the present sample (Cronbach’s α = 0.86).

### 2.5. Statistical Analysis

Descriptive statistics were used to summarize sociodemographic, clinical, and patient-reported variables. HRQoL differences between two groups were examined with independent-samples *t* tests, and differences across more than two groups were examined with one-way analysis of variance. Effect sizes were reported as Cohen’s d for two-group comparisons and eta squared (η^2^) for analysis-of-variance models. The association between sex and tumor site was examined using χ^2^ testing and Cramer’s V.

Pearson correlations were used to examine bivariate associations among symptom burden, depressive symptoms, resilient coping, and HRQoL. The primary model was a first-stage moderated mediation model estimated with IBM SPSS Statistics version 29.0 (IBM Corp., Armonk, NY, USA) and the PROCESS macro version 2.16.3 (Andrew F. Hayes). Symptom burden was specified as the predictor, depressive symptoms as the mediator, resilient coping as the moderator of the symptom burden–depressive symptoms association, and HRQoL as the outcome. Conditional indirect effects were estimated using 5000 bootstrap resamples and were evaluated at low, mean, and high levels of resilient coping. The index of moderated mediation was used to evaluate whether the indirect association varied significantly across levels of resilient coping.

Analyses were conducted using complete cases for the variables required in each model; no imputation was performed. As a sensitivity analysis, hierarchical linear regression examined whether associations between symptom burden, depressive symptoms, resilient coping, and HRQoL remained after adjustment for age, sex, and ECOG-PS. Because only baseline data were analyzed, mediation terminology refers to statistical decomposition of cross-sectional associations and not to temporal or causal mediation. Statistical significance was set at two-tailed *p* < 0.05.

## 3. Results

### 3.1. Sample Characteristics

A total of 207 patients from six centers were screened for eligibility, of whom 194 were included in the baseline sample. Comparisons between participants with complete and incomplete data showed no statistically significant differences in symptom burden, depressive symptoms, or HRQoL (all *p* > 0.05).

The cohort comprised 86 patients (44.3%) with breast cancer, 77 (39.7%) with gynecological tumors, and 31 (16.0%) with genitourinary cancer. Among gynecological cancers, ovarian (48%), cervical (28%), and endometrial (24%) cancers were the most frequent. The genitourinary group mainly comprised prostate (47%), kidney (31%), bladder (15%), and testicular (7%) cancers. The sample included 72.7% women and 27.3% men. Sex distribution differed significantly across tumor groups (χ^2^ = 75.57, *p* < 0.001; Cramer’s V = 0.62), indicating a strong association between sex and tumor site. Most participants were married or partnered (66.5%). Sociodemographic characteristics were otherwise relatively balanced across age, education, and employment categories.

Most patients had unresectable disease (89.2%) and metastatic stage IV disease (90.2%) (Table 1). Planned treatment was chemotherapy alone in 94 patients (48.5%), chemotherapy plus targeted therapy in 72 (37.1%), chemotherapy plus immunotherapy in 20 (10.3%), and other regimens in 8 (4.1%).

### 3.2. HRQoL According to Sociodemographic Characteristics

Mean EQ-VAS scores differed significantly by sex, with men reporting higher HRQoL than women (72.16 ± 15.9 vs. 65.52 ± 17.3; t = 2.424, *p* = 0.016; d = 0.39). Because sex and tumor site were strongly associated, this difference should be interpreted as descriptive rather than as evidence of an independent sex effect. Age did not reach conventional statistical significance, although patients aged ≥65 years had numerically higher EQ-VAS scores than younger patients (69.69 ± 17.4 vs. 65.02 ± 16.6; *p* = 0.058; d = −0.27). No significant differences in EQ-VAS scores were observed according to marital status, education, or employment status (all *p* > 0.05).

### 3.3. HRQoL According to Clinical and Tumor Characteristics

EQ-VAS scores did not significantly differ across the main clinical and tumor-related characteristics (Table 2). Mean scores were similar across tumor sites (breast: 65.40 ± 18.3; gynecological: 69.12 ± 16.9; genitourinary: 68.25 ± 14.8; F = 1.003, *p* = 0.369, η^2^ = 0.010). No significant differences were observed according to resectability, histology, disease stage, planned treatment type, ECOG-PS, or oncologist-estimated life expectancy. Patients with ECOG-PS ≥2 had numerically lower EQ-VAS scores than those with ECOG-PS 0–1, but the difference was not statistically significant (63.31 ± 22.1 vs. 67.90 ± 16.4; *p* = 0.222; d = 0.27).

### 3.4. Associations Among Symptom Burden, Depressive Symptoms, Resilient Coping, and HRQoL

**Figure 1:**
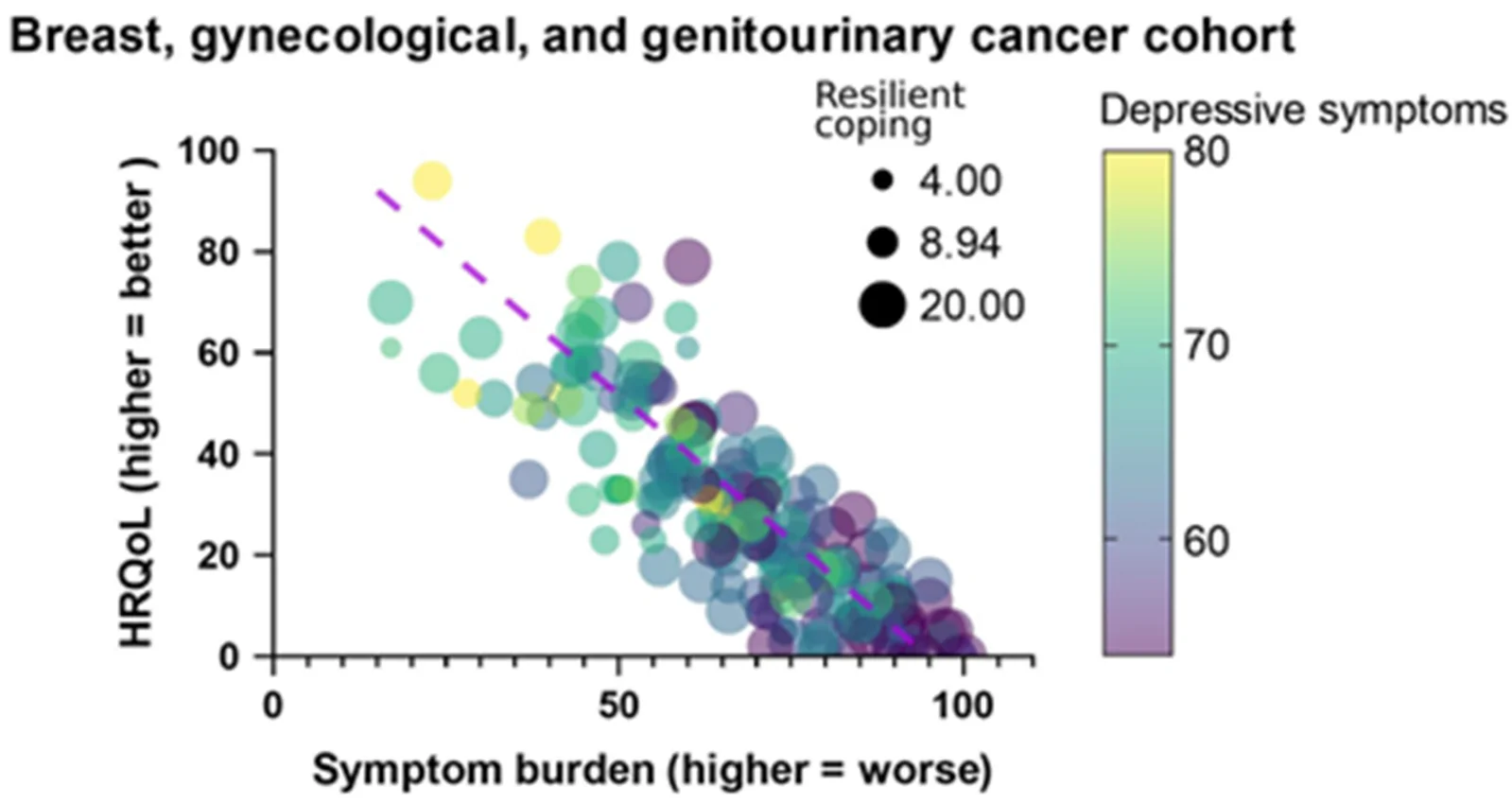
Association between symptom burden, depressive symptoms, resilient coping, and HRQoL. Higher color-scale values indicate greater depressive symptom severity, and point size represents resilient coping. Higher symptom burden and lower HRQoL indicate a less favorable patient-reported profile. The dashed line represents the fitted linear association between symptom burden and HRQoL.

Patient-reported symptom burden showed a strong inverse correlation with EQ-VAS scores (r = −0.827, *p* < 0.001), indicating that higher burden was associated with poorer global self-rated health. Resilient coping showed a modest positive correlation with HRQoL (r = 0.233, *p* = 0.001) and a moderate inverse correlation with depressive symptoms (r = −0.364, *p* < 0.001). Figure 1 displays the inverse pattern between symptom burden and HRQoL, with depressive symptom severity represented by color intensity and resilient coping by point size.

### 3.5. Moderated Mediation Model

The moderated mediation model was estimated using the full study sample (N = 194), with symptom burden as the predictor, depressive symptoms as the mediator, resilient coping as the first-stage moderator, and HRQoL as the outcome.

In the first-stage model, higher symptom burden was associated with higher depressive symptoms (B = 0.275, 95% CI 0.118 to 0.431, *p* < 0.001). The model explained 31.2% of the variance in depressive symptoms (R^2^ = 0.312; F = 28.53, *p* < 0.001). Resilient coping showed a non-significant inverse association with depressive symptoms (B = −0.307, *p* = 0.083), and the symptom burden × resilient coping interaction was not statistically significant (B = −0.009, *p* = 0.083).

In the outcome model, higher depressive symptoms (B = −0.529, 95% CI −0.743 to −0.316, *p* < 0.001) and higher symptom burden (B = −0.642, 95% CI −0.715 to −0.569, *p* < 0.001) were independently associated with poorer HRQoL. The model explained 72.1% of the variance in HRQoL (R^2^ = 0.721; F = 245.41, *p* < 0.001).

**Figure 2:**
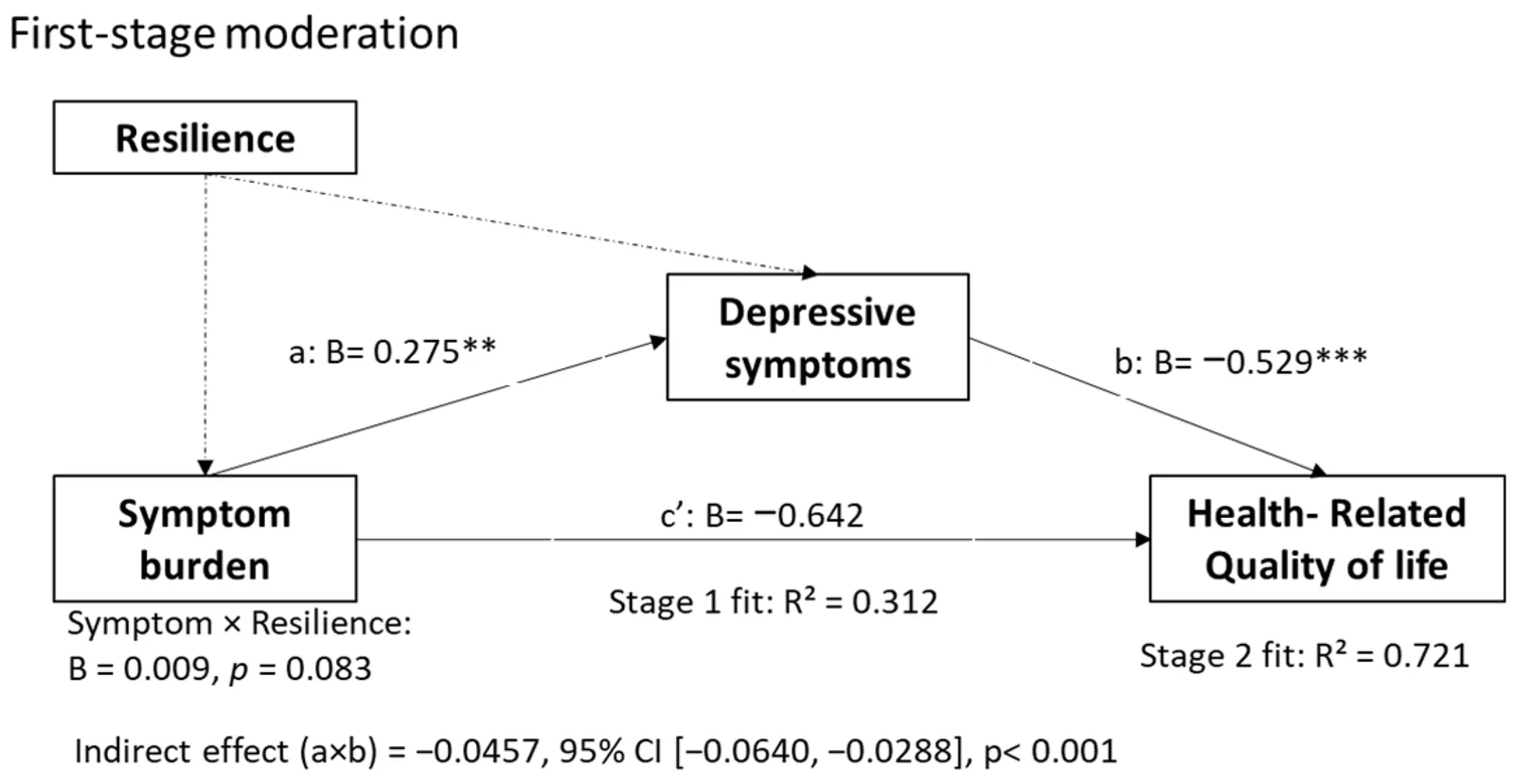
First-stage moderated mediation model linking symptom burden, depressive symptoms, and HRQoL, with resilient coping as a first-stage moderator. Unstandardized coefficients are shown. Solid arrows represent the estimated direct paths, and dashed arrows represent the moderating effect of resilient coping.

Bootstrap analyses showed negative conditional indirect effects of symptom burden on HRQoL through depressive symptoms at low resilient coping (B = −0.096, 95% bootstrap CI −0.165 to −0.053), mean resilient coping (B = −0.077, 95% bootstrap CI −0.120 to −0.047), and high resilient coping (B = −0.057, 95% bootstrap CI −0.100 to −0.028). However, the index of moderated mediation was not statistically significant (0.0049, 95% bootstrap CI −0.0006 to 0.0137), indicating insufficient evidence that resilient coping significantly changed the magnitude of the indirect association. Conditional indirect effects should therefore be interpreted as evidence of a statistical indirect association through depressive symptoms, not as evidence that resilient coping moderated that association (Figure 2 and Table S1).

In the sensitivity analysis, age, sex, and ECOG-PS explained 1.7% of the variance in HRQoL in the first step (R^2^ = 0.017; F = 1.10; *p* = 0.352). After adding symptom burden, depressive symptoms, and resilient coping, explained variance increased to 71.6% (ΔR^2^ = 0.699, *p* < 0.001). In the fully adjusted model, symptom burden (β = −0.694, *p* < 0.001), depressive symptoms (β = −0.192, *p* < 0.001), and resilient coping (β = 0.122, *p* < 0.001) remained independently associated with HRQoL, whereas age, sex, and ECOG-PS were no longer statistically significant predictors.

## 4. Discussion

In this multicenter pretreatment analysis, we simultaneously evaluated depressive symptoms as a statistical mediator and resilient coping as a potential moderator. Depressive symptoms were independently associated with lower HRQoL and statistically accounted for part of the association between symptom burden and HRQoL. Although resilient coping was associated with higher HRQoL and fewer depressive symptoms in bivariate analyses, it did not significantly moderate the symptom burden–depressive symptoms association. Conventional clinical and tumor-related characteristics showed limited associations with HRQoL. Future longitudinal analyses may further clarify these associations across more heterogeneous clinical settings.

The descriptive sex difference in EQ-VAS scores should be interpreted cautiously. Women reported lower HRQoL than men, but sex was closely linked to tumor site. Because breast, gynecological, and genitourinary cancers differ in symptom burden, treatment, and disease experience, the observed difference likely reflects the combined influence of sex and tumor context rather than an independent effect of sex [19,20,21]. Likewise, the absence of differences in global EQ-VAS scores across tumor sites does not imply similar domain-specific HRQoL, as comparable global ratings may mask distinct patterns of impairment. Future studies should incorporate tumor-specific HRQoL measures.

Clinical and tumor-related variables did not significantly distinguish HRQoL in this baseline cohort. This does not imply that stage, performance status, treatment plan, or prognosis are irrelevant to HRQoL. The sample was clinically selective: most patients had unresectable and metastatic disease, ECOG-PS variability was limited, and several subgroups were small. Prior evidence supports associations between functional status, advanced disease context, systemic inflammation, symptom burden, and clinical outcomes [22,23,24,25]. The present findings are more appropriately interpreted as indicating that, within this relatively homogeneous systemic-treatment cohort, patient-reported symptoms and depressive symptoms discriminated global HRQoL more strongly than the available clinical categories.

The statistical indirect association through depressive symptoms is clinically relevant but should not be read causally. Physical symptoms may contribute to depressive symptoms; depressive symptoms may intensify symptom perception and appraisal; and both may be influenced by disease context, social support, prior mental health history, inflammation, prognosis, and treatment expectations. The findings nevertheless support the clinical value of integrating psychological assessment with symptom monitoring at the pretreatment visit, rather than treating emotional distress as secondary to physical symptom management [4,5,6]. Consistent with previous oncology cohorts, symptom burden showed one of the strongest associations with global HRQoL. Although the inverse correlation observed in our cohort (r = −0.827) exceeded the correlations reported in breast cancer cohorts (approximately |ρ| = 0.5–0.6) [4], direct comparisons should be interpreted cautiously because symptom burden was derived from a composite score and HRQoL was assessed using a different global instrument. Nevertheless, because both measures were patient-reported and assessed concurrently, some conceptual overlap and shared method variance may have contributed to the strength of this association. Clinically, the close association between symptom burden, depressive symptoms, and self-rated health supports an integrated assessment of physical and emotional symptoms before systemic therapy. This approach is consistent with recent models emphasizing early, needs-based integration of supportive and palliative care to address multidimensional symptom burden and quality-of-life concerns in patients with advanced cancer [26,27]. Although causality cannot be inferred, pretreatment assessment may help identify patients with greater supportive care needs.

The findings for resilient coping refine, rather than negate, the role of adaptive coping resources. Higher BRCS scores were associated with more favorable HRQoL and lower depressive symptom, consistent with prior work on resilience and coping in oncology [7,8,9,10,28,29,30]. However, resilient coping did not significantly moderate the indirect association linking symptom burden, depressive symptoms, and HRQoL. One explanation is that resilient coping may be more relevant to longer-term adjustment than to the immediate pretreatment relationship between symptom burden and distress. Alternatively, high symptom burden may outweigh the protective effects of coping resources at this stage of the disease. Methodological factors may also have contributed, as interaction effects are typically smaller than main effects and require larger samples to detect reliably. Thus, limited statistical power cannot exclude a small but potentially meaningful moderating effect. In addition, the BRCS assesses only one dimension of adaptive coping. Therefore, the absence of a significant interaction should not be interpreted as evidence that resilient coping lacks clinical relevance. Longitudinal studies may clarify whether its influence becomes more apparent after treatment initiation.

### 4.1. Strengths and Limitations

Key strengths include the multicenter design, assessment before systemic therapy, the use of validated patient-reported outcome measures, and the explicit distinction between statistical indirect associations and causal mediation. In addition, symptom burden, depressive symptoms, and resilient coping were examined within an integrated model rather than as isolated correlates of HRQoL.

Several limitations should be acknowledged. First, although the parent study was prospective, the present analysis used only baseline data; therefore, temporal ordering and causal mediation cannot be inferred. Because this was a secondary analysis of the available baseline cohort, power to detect small interaction effects may have been limited; therefore, a small moderating effect cannot be excluded. Second, the cohort combined heterogeneous tumor sites and had uneven subgroup sizes, particularly for genitourinary cancers, limiting tumor-specific inference. Third, sex and tumor site were strongly associated, making it difficult to disentangle independent sex effects from tumor-context effects. Fourth, the EQ-VAS is a single global self-rated health measure and does not capture domain-specific HRQoL, including emotional, social, sexual, urinary, bowel, body image, or role functioning. Fifth, the symptom/problem burden score was derived from EORTC QLQ-C30 symptom scales/items and may not capture tumor-specific symptom domains assessed by disease-specific modules. Sixth, all PROs were self-reported at the same assessment, which may introduce recall effects, response style effects, and common-method variance. Seventh, the primary PROCESS model was unadjusted; although sensitivity analyses adjusted for age, sex, and ECOG-PS, information on potentially relevant variables such as psychiatric history, social support, socioeconomic status, inflammatory status, treatment intent, and prognosis was not available, and residual confounding cannot be excluded. Finally, the study was conducted in Spanish hospitals, and generalizability to other healthcare systems, cultural contexts, and treatment pathways should be assessed.

### 4.2. Clinical Implications

These findings support routine pretreatment assessment of patient-reported symptom burden and depressive symptoms in patients starting systemic therapy for breast, gynecological, or genitourinary cancers. Higher symptom burden and depressive symptom scores may indicate a need for further assessment and, when clinically appropriate, earlier symptom management, psycho-oncology or supportive care referral, or palliative care involvement. The absence of significant moderation by resilient coping does not support assuming that high resilient coping offsets symptom burden; however, small moderation effects cannot be excluded. Resilient coping may therefore be considered a complementary marker of adjustment, while symptom control and emotional distress remain primary targets of supportive oncology. These implications are most directly applicable to patients with advanced, predominantly metastatic disease who are about to start a systemic treatment regimen.

## 5. Conclusions

In patients with breast, gynecological, and genitourinary cancers assessed before initiation of systemic therapy, lower HRQoL was more strongly associated with patient-reported symptom/problem burden and depressive symptoms than with the available clinical or tumor-related characteristics. However, because most participants had metastatic, unresectable disease and clinical variability was limited, these null group comparisons should be interpreted cautiously. Depressive symptoms statistically accounted for part of the association between symptom burden and HRQoL, whereas resilient coping did not significantly moderate this pathway. These findings support early PRO-based assessment of symptoms and depressive symptoms in routine oncology care and justify longitudinal analyses to clarify temporal ordering and evaluate which patients are most likely to benefit from targeted supportive interventions.

## Figures and Tables

**Table 1:** Health-related quality of life according to sociodemographic variables.

Variable	Group	Mean	SD	t	*p*-Value	Effect Size
Sex	Men	72.16	15.9	2.424	0.016	d = 0.39
	Women	65.52	17.3			
Age	<65 years	65.02	16.6	−1.904	0.058	d = −0.27
	≥65 years	69.69	17.4			
Marital status	Married/partnered	66.38	16.7	−1.085	0.279	d = −0.17
	Not partnered	69.22	18.1			
Education level	Primary school or lower	67.98	16.6	0.481	0.631	d = 0.07
	Secondary education or higher	66.78	17.7			
Employment status	Unemployed/retired	66.85	17.4	−0.378	0.706	d = −0.05
	Employed	67.79	17.1			

Note. HRQoL was assessed using the EQ-VAS. Values are mean and standard deviation. Cohen’s d is shown for two-group comparisons; positive values indicate higher mean EQ-VAS in the first listed group.

**Table 2:** Health-related quality of life according to clinical and tumor characteristics.

Variable	Group	*n* (%)	Mean	SD	t/F	*p*-Value	Effect Size
Tumor site	Breast	86 (44.3)	65.40	18.3	1.003	0.369	η^2^ = 0.010
	Gynecological	77 (39.7)	69.12	16.9			
	Genitourinary	31 (16.0)	68.25	14.8			
Resectability	Unresectable	173 (89.2)	67.43	17.2	0.235	0.815	d = 0.05
	Resectable	21 (10.8)	66.50	17.9			
Histology	Adenocarc.	114 (58.8)	67.27	17.2	−0.059	0.953	d = −0.01
	Other	80 (41.2)	67.42	17.4			
Stage	Locally advanced	19 (9.8)	66.03	16.4	−0.346	0.729	d = −0.08
	Metastatic disease (IV)	175 (90.2)	67.47	17.3			
Treatment type	Chemotherapy	94 (48.5)	67.43	17.3	0.193	0.901	η^2^ = 0.003
	Chemotherapy + immunotherapy	20 (10.3)	68.00	15.3			
	Chemotherapy + targeted therapy	72 (37.1)	67.52	17.4			
	Other regimens	8 (4.1)	62.85	21.3			
ECOG-PS	0–1	170 (87.6)	67.90	16.4	1.224	0.222	d = 0.27
	≥2	24 (12.4)	63.31	22.1			
Oncologist-estimated life expectancy	<18 months	58 (29.9)	67.70	15.9	0.194	0.846	d = 0.03
	≥18 months	136 (70.1)	67.17	17.7			

Note. HRQoL was assessed using the EQ-VAS. ECOG-PS = Eastern Cooperative Oncology Group performance status; Adenocarc. = adenocarcinoma. Cohen’s d is reported for two-group comparisons and η^2^ for ANOVA models.

## Data Availability

The datasets generated and analyzed during the current study are not publicly available because of privacy and confidentiality restrictions. Anonymized data may be available from the corresponding author upon reasonable request and subject to applicable ethical and legal approvals.

## Supplementary Materials

The following supporting information can be downloaded at: https://www.mdpi.com/article/10.3390/curroncol33080482/s1. Table S1: Complete-case moderated mediation model for HRQoL: symptom burden → depressive symptoms → HRQoL, with resilient coping as a first-stage moderator.
