# Supplementary material for: Symptom Burden, Depressive Symptoms, Resilient Coping, and Pretreatment Health-Related Quality of Life with Breast, Gynecological and Genitourinary Cancer
Source: Curr Oncol. 2026 Aug 15;33(8):482. doi: 10.3390/curroncol33080482 (PMC13511463; doi:10.3390/curroncol33080482)
Supplement: Supplementary file 1 [file curroncol-33-00482-s001.zip › curroncol-4413750-supplementary.pdf]

Current Oncology

MDPI

Supplementary Material

Table S1. Complete-case moderated mediation model for HRQoL: symptom burden → depressive symptoms → HRQoL, with resilient coping as a first-stage moderator.

Panel A. First-stage model (Outcome: depressive symptoms)

Predictor / effect	B	SE	t	p	95% CI LL	95% CI UL
Intercept	64.059	2.693	23.79	<0.001	58.747	69.370
Symptoms/problem burden	0.275	0.080	3.45	<0.001	0.118	0.431
Resilient coping (BRCS)	−0.307	0.176	−1.75	0.083	−0.654	0.040
Symptoms × BRCS	−0.009	0.005	−1.74	0.083	−0.019	0.001

Model fit: R = 0.558, R^2 = 0.312, MSE = 32.10, F = 28.53, $p < 0.001$.

Panel B. Second-stage model (Outcome: HRQoL)

Predictor / effect	B	SE	t	p	95% CI LL	95% CI UL
Intercept	119.559	6.571	18.19	<0.001	106.597	132.521
Depressive symptoms (BSI-18)	−0.529	0.108	−4.88	<0.001	−0.743	−0.316
Symptoms/problem burden	−0.642	0.037	−17.32	<0.001	−0.715	−0.569

Model fit: R = 0.849, R^2 = 0.721, MSE = 83.96, F = 245.41, $p < 0.001$.

Panel C. Conditional indirect effects (bias-corrected bootstrap)

Moderator level (BRCS)	Indirect effect (B)	Boot SE	BootLLCI	BootULCI
Low BRCS (10.11)	−0.096	0.028	−0.165	−0.053
Mean BRCS (14.17)	−0.077	0.018	−0.120	−0.047
High BRCS (18.22)	−0.057	0.018	−0.100	−0.028

Conditional indirect effects are reported for conventional low, mean, and high levels of resilient coping.

Panel D. Index of moderated mediation (bias-corrected bootstrap)

Moderator level (BRCS)	Indirect effect (B)	Boot SE	BootLLCI	BootULCI
Index	0.0049	0.0036	−0.0006	0.0137

Notes. Unstandardized coefficients are reported. Two-tailed p-values are shown. Conditional indirect effects and the moderated mediation index are based on 5000 bootstrap resamples. BRCS = Brief Resilient Coping Scale; HRQoL = health-related quality of life.
